# LC3-associated phagocytosis: a sorting mechanism for ubiquitinated membrane proteins?

**DOI:** 10.1080/27694127.2022.2040765

**Published:** 2022-03-22

**Authors:** Femmy C. Stempels, Geert van den Bogaart

**Affiliations:** Department of Molecular Immunology, Groningen Biomolecular Sciences and Biotechnology Institute, University of Groningen, the Netherlands

**Keywords:** autophagy, inhibitor, LAP, LC3-associated phagocytosis, membrane domain

## Abstract

The process of LC3-associated phagocytosis (LAP) combines elements of phagocytosis and autophagy. LAP is involved in clearance of pathogens and cell debris, antigen presentation, and immune signaling. However, the mechanistic role of LAP is incompletely understood. Based on observations from our recent study, we propose that LAP might serve as a sorting mechanism to cluster proteins destined for degradation into discrete domains of the phagosomal membrane.

LAP is hallmarked by the formation of so-called LAPosomes, where Atg8-family protein member MAP1LC3B/LC3 directly conjugates to the single membrane of phagosomes. In this process, LC3 is converted from a soluble, cytosolic LC3-I form to the phosphatidylethanolamine (PE)-conjugated LC3-II form. LAPosomes have been commonly observed in immune phagocytes, such as macrophages and dendritic cells (DCs), for multiple phagocytic cargoes. Selectively targeting LAP or canonical autophagy will facilitate studies aimed at distinguishing the mechanisms and functional roles of these processes. In addition, it will aid translational research for treatment of LAP and autophagy-related diseases, including infectious and autoimmune diseases.

However, a problem is that the mechanisms of LAP and canonical autophagy are mostly shared, making it challenging to develop drugs that target one process without affecting the other. For example, both processes depend on lysosomal acidification, and drugs that target the V-ATPase can be expected to affect both. Moreover, the ubiquitin-like conjugation system that anchors LC3 to the target membrane is also shared between autophagy and LAP and hence cannot be used as a target to distinguish between these processes. Similarly, while the receptors that trigger LAP, such as toll-like receptors, C-type lectins, IgG receptors, and scavenger receptors, are not involved in autophagy, targeting these receptors will affect other important cellular processes, including inflammatory signaling, endo/phagocytosis, and antigen degradation. The protein RUBCN/rubicon regulates autophagy and LAP differently, as it inhibits autophagy but promotes LAP. However, although interfering with RUBCN might inhibit LAP, the increased levels of autophagy could mask or interfere with downstream effects.

To address this challenge, we searched for compounds that selectively inhibit autophagy, but do not affect LAP. We studied the effect of two new autophagy inhibitors on LAP: SAR405, which inhibits the phosphatidylinositol 3-kinase PIK3C3/VPS34, a critical regulator of both autophagy and LAP, and ethyl (2-(5-nitrothiophene-2-carboxamido) thiophene-3-carbonyl) carbamate (EACC), which inhibits STX17 (syntaxin 17)-mediated fusion between autophagosomes and lysosomes [1]. We compared these inhibitors with two canonical inhibitors of autophagy: the V-ATPase inhibitor bafilomycin A_1_ and the lysosomotropic base chloroquine. In human dendritic cells (DCs) loaded with zymosan particles, we found that bafilomycin A_1_ and SAR405 inhibit both autophagy and LAP. However, chloroquine and EACC seem to affect autophagy but not LAP: whereas chloroquine increases the cellular levels of LC3-II, as is well known, and EACC decreases the levels of total LC3, these compounds do not significantly affect LC3 recruitment to the phagosomes. Thus, chloroquine and EACC might be used to distinguish canonical autophagy from LAP.

However, our finding that EACC reduces the total cellular pool of LC3 in DCs was surprising, as this contrasted with previous findings in HeLa cells where EACC results in a reduction of only LC3-II, and not of LC3-I. In DCs, EACC still reduces levels of LC3 in the presence of bafilomycin A_1_, indicating that this reduction is not due to lysosomal degradation and hence unlikely to be related to its interference with STX17. Instead, co-incubation experiments with the proteasome inhibitor MG132 suggested that EACC promotes the breakdown of LC3 by the proteasome. Moreover, experiments with the ribosomal inhibitor cycloheximide reveal a fast turnover of LC3 in DCs, with ~80% of LC3 already being degraded within five hours even in absence of EACC. Hence, our data suggest that EACC promotes the degradation of LC3 by the proteasome, possibly via an off-target effect of EACC.

It has previously been shown that LC3 is degraded by the proteasome following its ubiquitination, and the fast turnover of LC3 in DCs might indicate that LC3 is rapidly ubiquitinated in DCs. Such rapid ubiquitination could help to explain another observation from our study: In our immunofluorescence microscopy experiments, we noticed a non-uniform localization of LC3 at the phagosomal membrane. Compared to immunolabeled CYBB/gp91^ph^°^x^, a phagosomal membrane protein, LC3 clusters at discrete punctate structures at the phagosomal membrane (Fig. 1A). Ubiquitinated proteins are delivered to the proteasome by receptor proteins, such as SQSTM1/p62. SQSTM1 non-covalently binds to the ubiquitin moiety by its UBA domain and SQSTM1 can also directly interact with LC3 with its LC3-interacting region (LIR) motif. SQSTM1 interacts with the proteasome via its PB1 domain. However, the PB1 domain also enables oligomerization of SQSTM1. This oligomerization is well known to result in aggregation of ubiquitinated proteins in cells with defects in autophagy. It therefore seems possible that SQSTM1, and/or other receptor proteins, clusters (ubiquitinated) LC3 and ubiquitinated proteins at the phagosomal membrane, which would result in the observed punctate structures. Supporting this, we observe both ubiquitin and SQSTM1 localizing at phagosomes in DCs.

**Figure 1.**
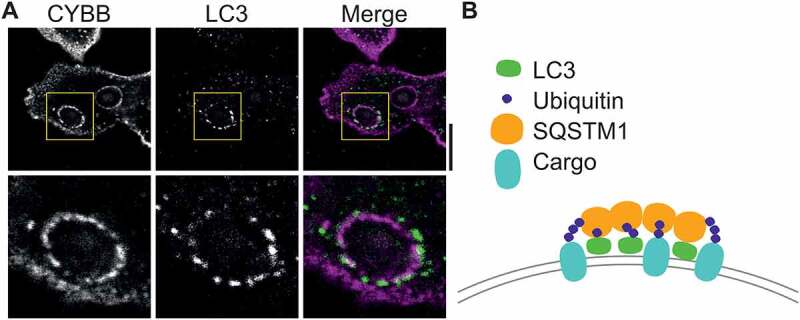
Unproven hypothesis for clustering of membrane proteins by LC3. (**A**) Confocal micrograph of human dendritic cell with ingested zymosan particles, and immunostaining for CYBB (magenta in merge) and LC3 (green). Image was taken from a published dataset [1]. Note the non-uniform, punctate appearance of LC3 at the surface of the LAPosome. Scale bar: 10 µm. (**B**) Hypothesis figure: clustering of (ubiquitinated) LC3 together with ubiquitinated cargo proteins by SQSTM1 receptor protein in the membrane of the LAPosome.

Interestingly, based on our own observations and the examination of published microscopy images, overexpressed GFP-tagged LC3 does not seem to locate in these punctate structures, but instead forms a ring at the phagosomal membrane. Perhaps the clustering of GFP-tagged LC3 is prevented due to the elevated expression levels or due to steric interference by the GFP tag.

An open question is, what is the function of the punctate structures? It is tempting to speculate that by the formation of these structures, LC3 and SQSTM1 (and/or other receptor proteins) cluster ubiquitinated cargo proteins that are destined for degradation into discrete regions of the phagosomal membrane (Fig. 1B). This clustering might facilitate the degradation of these proteins, as it could enhance their sorting into intralumenal vesicles by the endosomal sorting complex required for transport (ESCRT) machinery. Alternatively, the LC3-enriched membrane regions might somehow be pinched off from the phagosomal membrane into the cytosol, and subsequently be degraded by canonical autophagy. While purely speculative, such a mechanism would allow cells to efficiently degrade a subset of their phagosomal membrane proteins, which could be important for the functions of LAP.
